# The combination of HTATIP2 expression and microvessel density predicts converse survival of hepatocellular carcinoma with or without sorafenib

**DOI:** 10.18632/oncotarget.2019

**Published:** 2014-05-27

**Authors:** Wen-Quan Wang, Liang Liu, Hua-Xiang Xu, Hui-Chuan Sun, Chun-Tao Wu, Xiao-Dong Zhu, Wei Zhang, Jin Xu, Chen Liu, Jiang Long, Quan-Xing Ni, Zhao-You Tang, Xian-Jun Yu

**Affiliations:** ^1^ Department of Pancreatic and Hepatobiliary Surgery, Fudan University Shanghai Cancer Center; Department of Oncology, Shanghai Medical College, Fudan University; and Pancreatic Cancer Institute, Fudan University, Shanghai, China; ^2^ Liver Cancer Institute, Zhongshan Hospital, Fudan University, Key Laboratory for Carcinogenesis & Cancer Invasion, Chinese Ministry of Education, Shanghai, China; ^3^ Department of Hepatobiliary Surgery, Tianjin Medical University Cancer Institute and Hospital, Key Laboratory of Cancer Prevention and Therapy, Tianjin, China

**Keywords:** HTATIP2, microvessel density, sorafenib, hepatocellular carcinoma, prognosis

## Abstract

Our previous studies have demonstrated that sorafenib can promote the dissemination of hepatocellular carcinoma (HCC) through downregulation of HTATIP2, a suppressor of tumor growth and metastasis that is associated with inhibition of angiogenesis. Here, we investigated the predictive values of the HTATIP2 level and microvessel density (MVD) with or without sorafenib administration for HCC. Three independent cohorts were included. Using tissue microarray, we assessed the relationship between HTATIP2 expression/MVD and overall survival. The results showed that high HTATIP2 expression and a low MVD value were independent protective prognostic factors after curative HCC resection (297 cases/cohort 1); however, both parameters were converted to independent negative prognostic indicators for patients with postsurgical sorafenib treatment (69/143 cases/cohort 2; *P*<0.05 for all). This same relationship was observed in patients that received sorafenib treatment for advanced HCC (83 cases/cohort 3; efficacy measures and survival analyses, P<0.05 for all). Moreover, the combination of HTATIP2 and MVD had better power to predict patient death and disease recurrence (P<0.001 for both). We conclude that the combination of HTATIP2 and MVD predicts the converse survival of HCC with or without sorafenib intervention. Our findings can assist in the selection of candidates for personalized treatment with sorafenib.

## INTRODUCTION

Hepatocellular carcinoma (HCC) is the sixth most prevalent cancer globally and the third most common cause of cancer-related death[1]. The overall survival (OS) rate remains poor, although progress has been made recently[2]. Surgical resection of early-stage HCC is the most widely adopted therapy, and a 5-year survival of ~50% can be achieved[2]; however, the rate of postoperative recurrence or metastasis still remains high. Sorafenib, a receptor tyrosine kinase inhibitor (RTKI) that targets both tumor and endothelial cells, has improved the prognosis for patients with advanced HCC[3, 4]. Recently, the use of sorafenib has been investigated for the prevention of postsurgical recurrence and metastasis[5]. Despite endeavors to improve the efficacy of sorafenib, the survival benefit of this agent was found to be only a few months, probably due to the lack of effective tools that can assist in patient selection and predict individual outcomes.

**Table 1:** Patient characteristics

Characteristics	Cohort 1	Cohort 2		Cohort 3
	Non-sorafenib (n = 297)	Sorafenib (n = 69)	Non-sorafenib (n = 74)	Sorafenib (n = 83)
Age (years, median [range])	52 (22−80)	55 (28−75)	55 (32−76)	52 (18−75)
Gender (male/female)	248/49	61/8	62/12	77/6
Hepatitis B history (yes/no)	255/42	58/11	56/18	69/14
Hepatitis B e antigen (positive/negative)	113/184	22/47	14/60	22/61
Preoperative ALT (U/L, median [range])	42 (9−208)	44 (5−184)	51 (8−272)	35 (2−187)
α-Fetoprotein (ng/dl, median [range])	164 (0−60500)	248 (0−60500)	256 (0−60500)	196 (0−60500)
Liver cirrhosis (yes/no)	228/69	61/8	60/14	64/19
Tumor size (cm, mean ± SD)	5.57 ± 3.93	5.32 ± 4.41	5.37 ± 3.88	6.73 ± 4.91
Tumor differentiation (high/low)	209/88	26/43	41/33	NE
Tumor number (multiple/single)	39/258	20/49	11/63	68/15
Intrahepatic metastasis (yes/no)	43/254	34/35	32/42	NE
Tumor encapsulation (complete/no)	145/152	28/41	22/52	NE
Microvascular invasion (yes/no)	119/178	30/39	24/50	49/34
UICC TNM stage (I/II/IIIA)	36/128/133	10/39/20	8/25/41	NE

Abbreviations: ALT, alanine aminotransferase; SD, standard deviation; NE, not evaluated; UICC, International Union Against Cancer Classification; TNM, tumor-node-metastasis.

Cumulative studies have provided evidence and insight that sorafenib and another RTKI, sunitinib, can accelerate the spread of cancer in certain situations[6-9], suggesting that only some patients may benefit from these agents. Our previous studies have also demonstrated that sorafenib promotes invasiveness and metastasis of HCC through downregulation of HIV-1 Tat interactive protein 2 (HTATIP2) using animal tumor models[10, 11]; nonetheless, the clinical significance of the latter finding has not been fully elucidated. HTATIP2 plays an important role in the suppression of hepatocarcinoma growth and metastasis[12, 13], and may be associated with inhibition of angiogenesis[14]; however, the correlation between HTATIP2 expression and microvessel density (MVD) remains unclear. Moreover, the function of HTATIP2 as a prognostic factor after curative resection of HCC needs to be clarified.

Herein, we aimed to investigate the prognostic and/or predictive characteristics of HTATIP2 and MVD, separately and combinatorially, for survival of HCC patients in the presence and absence of sorafenib.

## RESULTS

### Patterns of HTATIP2 expression and microvessel distribution

**Figure 1:**
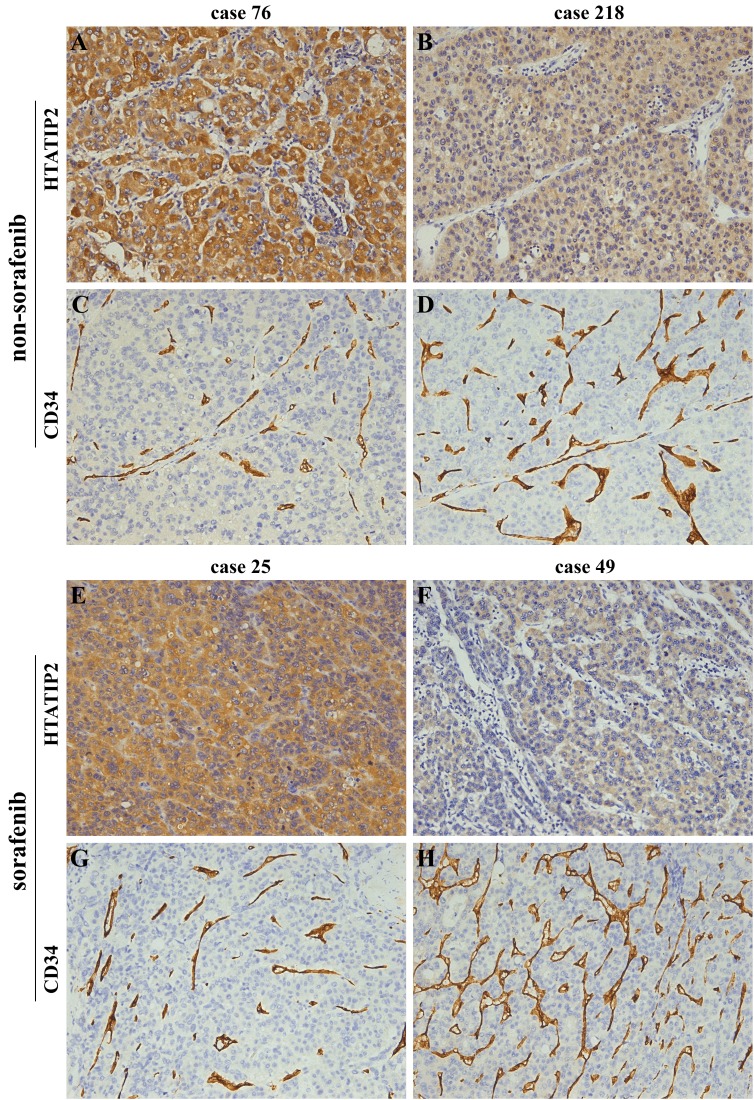
Images from representative samples with high or low HTATIP2 expression and microvessel density (MVD, indicated by CD34) as determined by immunostaining of tissue microarrays (A and C) Case 76 in cohort 1 and (E and G) case 25 in cohort 2 showed high HTATIP2 but low MVD; by contrast, (B and D) case 218 in cohort 1 and (F and H) case 49 in cohort 2 showed low HTATIP2 but high MVD (×200).

Immunostaining of HTATIP2 was mainly distributed in the cytoplasm of tumor cells or hepatocytes (data not shown). Most of the stromal cells were negative staining; although, sporadic positive staining on them was also observed (Figure 1A, B, E, F; supplemental Figure S1A and B). Specific staining of capillary-like vessels by anti-CD34 was also observed (Figure 1C, D, G, H; Figure S1C and D) in agreement with a previous study[15]. Most of the patients with strong positive HTATIP2 staining exhibited a relatively low MVD and vice versa. The average levels of HTATIP2 and MVD are listed in the footnotes of Table 2 and Supplemental Tables S1 and S5. We used the median value of HTATIP2 density or MVD as the cutoff points for the definition of the subgroups (high- versus low-risk groups)[10, 16], and these values were as follows: 0.0457/cohort 1, 0.0594/cohort 2, and 0.0626/cohort 3 for HTATIP2 and 0.0877/cohort 1, 0.0736/cohort 2, and 0.0756/cohort 3 for MVD. The HTATIP2 density was negatively correlated with MVD remarkably in the three cohorts (*r*=–0.279, *P*<0.001 for cohort 1; *r*=–0.231, *P*=0.006 for cohort 2; and *r*=–0.350, *P*=0.001 for cohort 3; Figure 1 and Figure S1).

**Table 2:** Relationship between intratumoral HTATIP2 expression/microvessel density and clinicopathological features in sorafenib non-administered cohort 1 after surgery

Variables	HTATIP2 densitya					Microvessel densitya				
	Low (n = 148)		High (n = 149)		P	Low (n = 148)		High (n = 149)		P
	No. of patients	%	No. of patients	%		No. of patients	%	No. of patients	%	
Age, yearsb	52.53 ± 11.41		51.81 ± 10.64		.570	51.10 ± 9.69		53.23 ± 12.13		.096
Gender					.263					.263
Male	120	81	128	86		120	81	128	86	
Female	28	19	21	14		28	19	21	14	
Hepatitis B history					.329					.048
Yes	130	88	125	84		133	90	122	82	
No	18	12	24	16		15	10	27	18	
HBeAg					.941					.081
Positive	56	38	57	38		49	33	64	43	
Negative	92	62	92	62		99	67	85	57	
ALT, U/Lb	60.14 ± 72.17		53.69 ± 42.18		.348	61.46 ± 75.67		52.38 ± 35.27		.185
AFP, ng/dlb	7818.98 ± 17501.52		3266.13 ± 10386.50		.007c	3451.37 ± 10501.47		7604.42 ± 17444.87		.014c
Liver cirrhosis					.916					.472
Yes	114	77	114	77		111	75	117	79	
No	34	23	35	23		37	25	32	21	
Tumor size, cmb	6.70 ± 4.53		4.45 ± 2.81		.000c	4.37 ± 2.44		6.77 ± 4.69		.000c
Tumor differentiation					.829					.082
High (Stage I–II)	105	71	104	70		111	75	98	66	
Low (Stage III–IV)	43	29	45	30		37	25	51	34	
Intrahepatic metastasis					.002					.014
Yes	31	21	12	8		14	9	29	19	
No	117	79	137	92		134	91	120	81	
Tumor encapsulation					.092					.863
Complete	65	44	80	54		73	49	72	48	
No	83	56	69	46		75	51	77	52	
Microvascular invasion					.022					.002
Yes	69	47	50	34		46	31	73	49	
No	79	53	99	66		102	69	76	51	
TNM stage					.066					.032
I	13	9	23	15		23	15	13	9	
II	60	40	68	46		69	47	59	39	
IIIA	75	51	58	39		56	38	77	52	

aThe densities of HTATIP2 and microvessel (CD34) were represented as the index of the integrated optical density/total area and area with positive staining/total area, respectively. The HTATIP2 density (mean ± standard deviation) was 0.0691 ± 0.0703 (median, 0.0457; range, 0.000024–0.389), and the microvessel density was 0.116 ± 0.105 (median, 0.0877; range, 0.00102–0.545).

bStudent's t-test, mean ± standard deviation.

cEqual variances not assumed.

P<0.05 was deemed to be statistically significant.

Abbreviations: HTATIP2, HIV-1 Tat interactive protein 2, 30 kDa; HBeAg, hepatitis B e antigen; ALT, alanine aminotransferase; AFP, α-fetoprotein; TNM, tumor-node-metastasis.

### Correlations between HTATIP2 expression/microvessel density and clinicopathological features

As shown in Table 2 of cohort 1, patients with a low intratumoral HTATIP2 expression or a high MVD were prone to exhibit large tumor size, high serum α-fetoprotein concentration, high tumor-node-metastasis (TNM) stage (borderline significance for HTATIP2), and the presence of intrahepatic metastasis and microvascular invasion. These correlations were verified in Supplemental Table S1 of cohort 2, which comprised 69 sorafenib-administered cases as well as 74 sorafenib non-administered cases. No relationship was found between HTATIP2 expression/MVD and other clinicopathological factors.

### Converse prognostic value of HTATIP2 expression/microvessel density on postoperative survival and recurrence between sorafenib non-administered and administered cohorts

**Figure 2:**
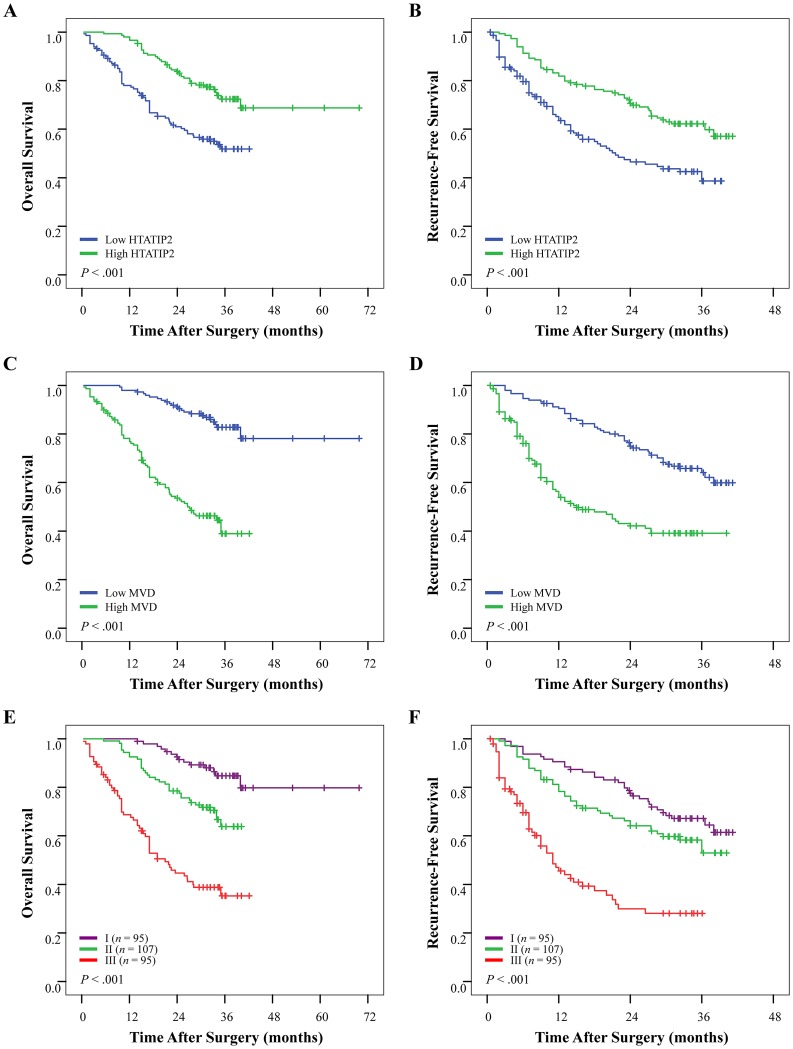
Cumulative overall survival (OS) and recurrence-free survival (RFS) curves of patients with high or low HTATIP2 density and microvessel density (MVD) as well as their combination in cohort 1 (see Results for details) Patients without sorafenib administration, who possessed (A and B) high HTATIP2 expression or (C and D) low MVD tended to have prolonged OS and RFS. (E and F) The combination of high HTATIP2 expression and low MVD predicted the best survival.

**Figure 3:**
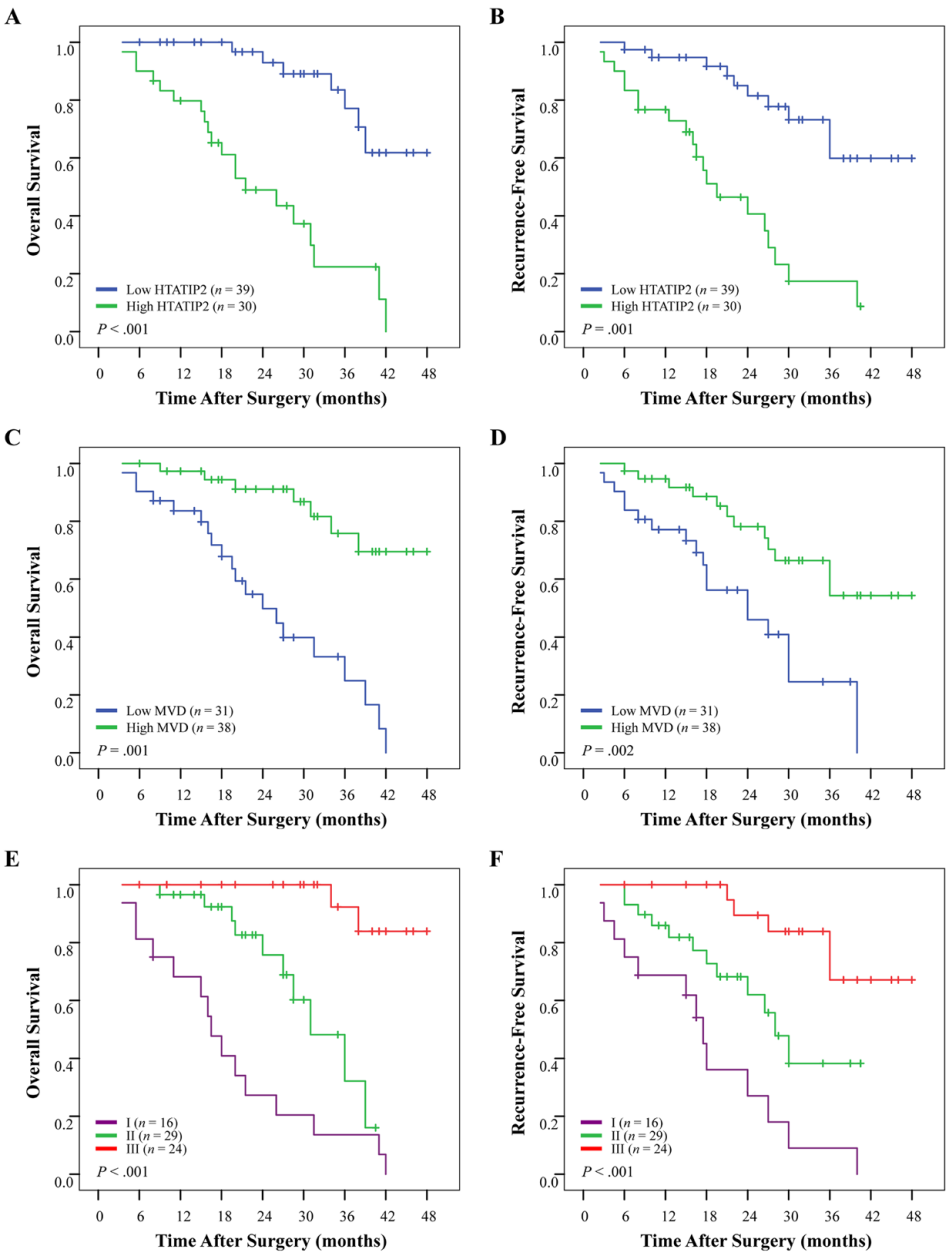
Cumulative overall survival (OS) and recurrence-free survival (RFS) curves of 69 sorafenib-administered patients with high or low HTATIP2 density and microvessel density (MVD) as well as their combination in cohort 2 (A and B) High HTATIP2 and (C and D) low MVD were associated with shortened OS and RFS. (E and F) The combination of high HTATIP2 and low MVD indicated the worst survival.

Univariate analyses of factors in the sorafenib non-administered cohort 1 revealed that tumor size, tumor number, tumor differentiation, presence of intrahepatic metastasis or microvascular invasion, and TNM stage were associated with OS and recurrence-free survival (RFS). Positive serum hepatitis B e antigen was also associated with RFS (Table 3). The median OS and RFS times were 34.1 months and 31.0 months, respectively, for patients with high HTATIP2 density and were significantly longer than that for patients with low HTATIP2 density (23.5 months and 13.0 months, respectively; *P*<0.001 for both; Figure 2A and B). By contrast, patients with a high MVD had a poor OS and RFS (*P*<0.001 for both; Figure 2C and; Table 3). However, in univariate analysis of factors for 69 sorafenib-administered patients in cohort 2, almost none of these clinicopathological features were related to OS and RFS, except that α-fetoprotein was associated with OS (Supplemental Table S2). In contrast to cohort 1, patients with high HTATIP2 expression or a low MVD had an even worse OS (*P*<0.001 and *P*=0.001, respectively) and RFS (*P*=0.001 and *P*=0.002, respectively) than those with low HTATIP2 expression or high MVD (Figure 3A–D; Table S2).

**Table 3:** Univariate and multivariate analyses for survival and recurrence in cohort 1

Factors	Overall survival				Recurrence-free survival			
	Univariate P	Multivariate			Univariate P	Multivariate		
		HR	95% CI	P		HR	95% CI	P
Age: ≤ 50 vs > 50 years	.150			NA	.604			NA
Gender: female vs male	.430			NA	.320			NA
Hepatitis B history: no vs yes	.219			NA	.915			NA
HBeAg: negative vs positive	.106			NA	.004	1.554	1.092–2.212	.014
Liver cirrhosis: no vs yes	.076			NA	.151			NA
ALT: ≤ 75 vs > 75 U/L	.716			NA	.391			NA
AFP: ≤ 300 vs > 300 ng/dl	.088			NA	.055			NA
Tumor size: ≤ 5 vs > 5 cm	< .001	2.565	1.666–3.948	< .001	< .001	1.469	1.013–2.129	.042
Tumor differentiation: low vs high	.001	1.706	1.134–2.566	.010	.010			NS
Tumor number: single vs multiple	.013			NS	.001			NS
Intrahepatic metastasis: no vs yes	< .001	1.804	1.137–2.861	.012	< .001	2.055	1.306–3.232	.002
Tumor encapsulation: no vs complete	.098			NA	.288			NA
Microvascular invasion: no vs yes	< .001			NS	< .001			NS
TNM stage: I vs II vs IIIA	< .001	1.663	1.128–2.453	.010	< .001	1.724	1.262–2.355	.001
HTATIP2 density: low vs high	< .001	0.841	0.380–0.986	.001	< .001	0.676	0.367–0.881	.039
Microvessel density: low vs high	< .001	4.084	2.560–6.514	< .001	< .001	2.361	1.621–3.439	< .001
Combine HTATIP2 and microvessel density	< .001			NA	< .001			NA

Abbreviations: HR, hazard ratio; CI, confidence interval; NA, not adopted; HBeAg, hepatitis B e antigen; ALT, alanine aminotransferase; AFP, α-fetoprotein; NS, not significant; TNM, tumor-node-metastasis; HTATIP2, HIV-1 Tat interactive protein 2.

Sorafenib prolonged postoperative OS and RFS compared with the control (*P*=0.008 and P=0.009, respectively; Figure S2A and B). Next, we classified cohort 2 into two subgroups with either high or low expression of HTATIP2 according to the HTATIP2 density. The analyses showed that sorafenib did not impact patient outcome in the HTATIP2 high-expression group (OS, *P*=0.191 and RFS, *P*=0.617; Figure S2C and D); however, in the HTATIP2 low-expression group OS and RFS were dramatically prolonged (*P*<0.001 and *P*=0.001, respectively; Figure S2E and F).

Risk factors identified by univariate analysis of cohorts 1 and 2 were pooled into a multivariate Cox proportional hazards analysis (Table 3; Table S2). Both high HTATIP2 expression and low MVD were independent protective factors of OS (hazard ratio [HR]=0.841, *P*=0.001 and HR=4.084, *P*<0.001, respectively) and of RFS (HR=0.676, *P*=0.039 and HR=2.361, *P* <0.001, respectively) for cohort 1. Unexpectedly, both biomarkers were independent risk factors of OS (HR=4.567, *P*=0.001 and HR=0.254, *P*=0.003, respectively) and of RFS (HR=4.165, *P*<0.001 and HR=0.444, *P*=0.034, respectively) for sorafenib-administered patients in cohort 2.

### Prediction of the combination of HTATIP2 with microvessel density on postoperative survival and receiver operating characteristic (ROC) analyses

Patients (cohort 1 and 69 sorafenib-administered patients in cohort 2) were first categorized into three groups according to their HTATIP2 density and MVD: group I, high HTATIP2 and low MVD; group II, high HTATIP2 and high MVD as well as low HTATIP2 and low MVD; and group III, low HTATIP2 and high MVD. When performing the ROC analysis, group II was further divided into two groups with both high/low levels of the two biomarkers. The prognostic analysis of both cohorts showed significant differences in both OS and RFS among the three groups (*P*<0.001 for all; Table 3; Table S2). The cumulative OS and RFS rates of group I were the best for cohort 1 (Figure 2E and F) but were the worst for sorafenib-administered patients of cohort 2 (Figure 3E and F); the converse case was observed for group III.

Clinicopathological factors identified by multivariate analysis and the combination of HTATIP2 expression and MVD were included, and their predictive values were studied by ROC analysis (see Supplemental Materials and Methods for details). HTATIP2 expression, MVD, and the combination of both parameters precisely predicted death and recurrence for both cohorts (*P*<0.05 for all), and the predictive value of the combinatorial group was the best among all the adopted factors (Figure S3). The area under the curve of this combination was 0.730/0.848 for death and 0.690/0.754 for recurrence in cohort 1/sorafenib-administered patients of cohort 2 (*P*<0.001 for all), respectively, and was greater than other factors (Tables S3 and S4).

### Prediction of HTATIP2 expression/microvessel density for patient survival to sorafenib in advanced HCC

**Figure 4:**
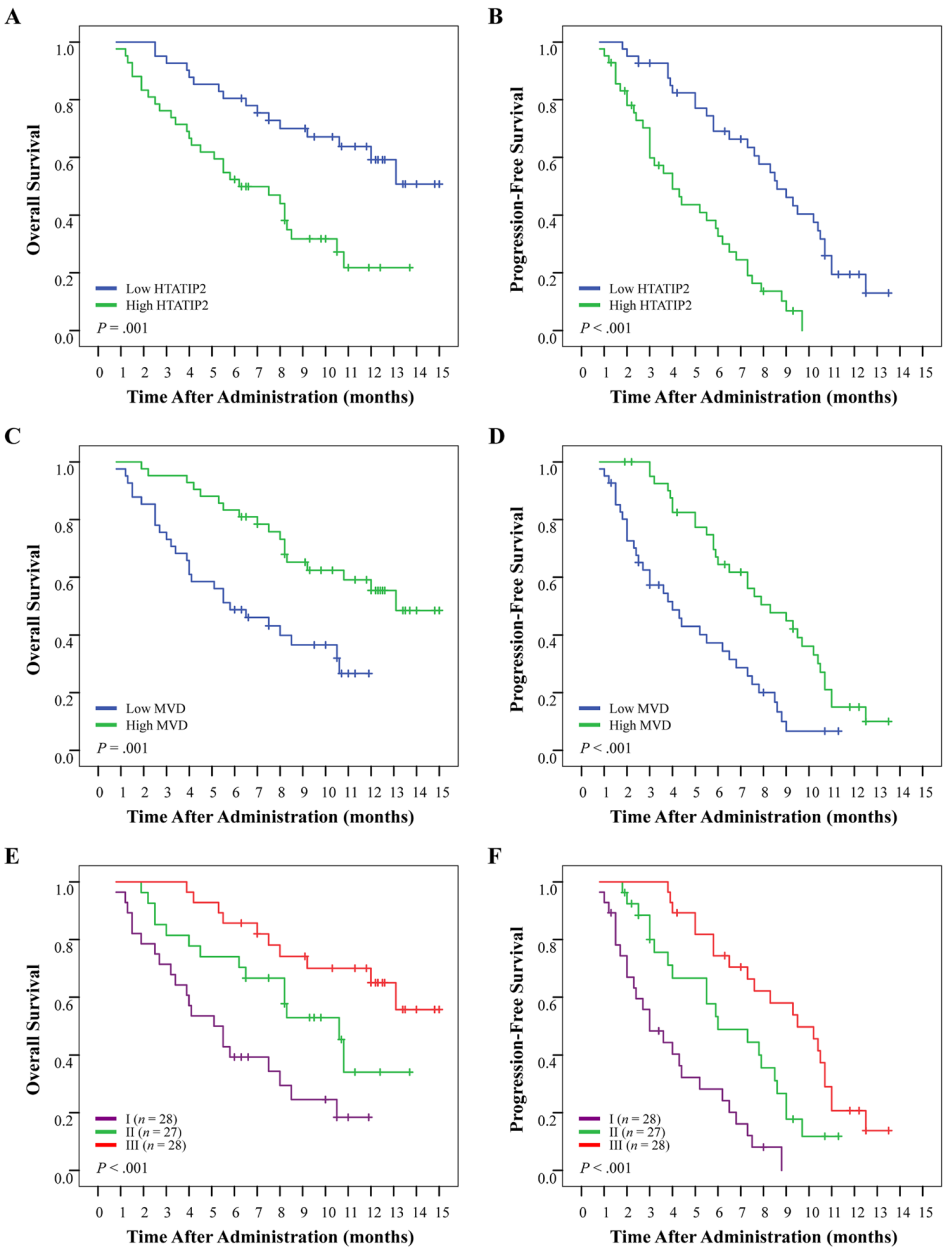
Cumulative overall survival (OS) and progression-free survival (PFS) curves of 83 sorafenib-administered patients with advanced-stage hepatocellular carcinoma Patients were classified into the high- or low-HTATIP2 expression group and into the high- or low-MVD group according to HTATIP2 density and MVD value, respectively; these subgroups were recombined into three groups for further analyses (see Results for details). (A and B) High HTATIP2 and (C and D) low MVD were associated with poor OS and PFS. (E and F) The combination of high HTATIP2 and low MVD suggested the shortest OS and earliest disease progression.

The classification of patients in cohort 3 was the same as that described above. Patients with high HTATIP2 expression or low MVD tended to have poor prognosis compared with those with low HTATIP2 or high MVD (both *P*=0.001 for OS and both *P*<0.001 for PFS; Figure 4A–D). The median OS and PFS were >13.0 and 8.6 months for the low-HTATIP2 group, but were only 6.2 and 4.0 months for the high-HTATIP2 group, respectively. In addition, the median OS and PFS were 13.1 and 8.3 months for the high MVD group, but were only 5.8 and 4.0 months for the low-MVD group, respectively. The combination of high HTATIP2 and low MVD predicted the worst OS and PFS (*P*<0.001 for both; Figure 4E and F). The disease-control rate was 43.4% for the low-HTATIP2 group and 28.9% for the high-HTATIP2 group (*P*=0.004). The disease-control rate was 42.2% for the high-MVD group and 30.1% for the low-MVD group (*P*=0.037; Table S5).

## DISCUSSION

In the present study, we found that tumor MVD was negatively correlated with the expression of HTATIP2. In contrast to MVD, high HTATIP2 expression in HCC was an independent protective prognostic factor after curative resection and was associated with small tumor size, a lower rate of intrahepatic metastasis and microvascular invasion, and much better prognosis. However, both high HTATIP2 expression and low MVD became independent risk factors when patients were treated with sorafenib as a postsurgical adjuvant therapy. Moreover, the combination of high HTATIP2 expression with low MVD predicted the best outcome for patients without sorafenib administration, but predicted the worst outcome for those administered sorafenib, compared with the two markers alone and other clinicopathological factors. Furthermore, these findings were confirmed using an independent cohort that included patients with advanced HCC.

HTATIP2, also known as 30-kilodalton HIV-1 Tat interacting protein (TIP30) or CC3, typically functions as a tumor suppressor and was initially identified in the highly metastatic human variant small cell lung carcinoma (SCLC) in comparison with the less metastatic classic SCLC cell lines[17]. HTATIP2 frequently exhibits downregulation in various tumor cells, such as breast cancer, colon cancer, pancreatic cancer, melanoma, glioblastoma, neuroblastoma, SCLC, and HCC cells[14, 17-22]. Studies in *HTATIP2*-deficient mice showed a dramatically increased susceptibility to tumorigenesis, including that of HCC[21, 23]. Other studies have shown that HTATIP2 inhibits human HCC cell growth and metastasis, as well as induce apoptosis, *in vitro* and *in vivo* [12, 13, 24]. In a previous study, we validated the suppressive role of HTATIP2 on HCC cells, a function that was related to the inhibition of the epithelial-to-mesenchymal transition (EMT) [10]. In the present study, we further revealed that patients with relatively high HTATIP2 expression tended to have a small tumor volume, diminished metastases, and prolonged postoperative survival, and these findings were consistent with those from previously described experimental research and with clinical findings from other tumor types[18, 23]. Here, we also described a negative correlation between HTATIP2 expression and MVD, implying that the putative antiangiogenic property plays a crucial role in the tumor inhibitory effects of HTATIP2[14, 22]. Consequently, the combination of high HTATIP2 expression and low MVD may predict the best survival after surgery.

Nevertheless, our results further showed that the combination of high HTATIP2 and low MVD predicted the worst survival when patients were adjunctively treated with sorafenib. Randomized trials of sorafenib have shown survival benefits for individuals with various tumors[3, 25] (ClinicalTrials.gov), although prometastatic side effects have also been observed[26]. Ebos et al. and Paez-Ribes et al. first reported the adverse results of antiangiogenic therapy in their experimental studies[6, 7]. Investigations of the underlying mechanism have focused on the host environment, tumor microenvironment, and tumor cells[27-29]. Among them, tumor hypoxia and impairment of vascular integrity were considered to be the two most important factors contributing to the prometastatic effects of antiangiogenic therapy[7, 9]. For example, we previously report that tumor-associated macrophages are recruited by sorafenib and contribute to the malignancy of HCC in association with hypoxia[30]; however, we have not detected predictive value of tumor-associated macrophages for sorafenib in our preliminary investigations (data not shown). In another previous study, we found that sorafenib directly downregulated HTATIP2 in tumor cells and provoked liver micrometastases[10]. This was the first report showing that sorafenib directly promoted invasiveness of HCC cells, and we demonstrated its clinical significance in the present study. Considering the critical role of HTATIP2 in the suppression of HCC growth and metastasis and the inhibition of proangiogenic capability of the tumor cells, we speculated that the invasive and metastatic potential of residual tumor cells with high HTATIP2 expression would be stimulated after downregulation of HTATIP2 expression following sorafenib treatment. Interestingly, the time to relapse/progression and overall survival were substantially shortened. Conceivably, in patients with high HTATIP2 expression, the efficacy of sorafenib application would not be expected, suggesting that patients with lower HTATIP2 expression are better candidates for sorafenib therapy.

Intriguingly, in the present study, in sorafenib-treated patients, we also found a reverse good prognosis for those with high tumor MVD. On the one hand, the cause may be that high MVD significantly correlates with low HTATIP2 density and that the low HTATIP2 expression enhanced the patients' sensitivity to sorafenib. On the other hand, patients with high MVD were more likely to have a higher sensitivity to antiangiogenic agents, such as sorafenib. We presume that this relationship was a result of the combined efficacy of both factors.

In conclusion, our results signify that the combination of HTATIP2 and MVD predicts the converse survival of HCC with or without sorafenib intervention and that patients with high HTATIP2 expression and low MVD level may not benefit from this drug. This finding can be used for the selection of candidates for personalized treatment with sorafenib. To date, given that no molecular biomarkers have been found that can predict the outcome of sorafenib treatment, our findings offer new hope for this unexplored avenue and lay the foundation for further translation in a prospective study.

## MATERIALS AND METHODS

### Patient selection:

Three independent cohorts (Table 1) were included in the present study. In cohort 1, 297 patients who underwent curative liver resection for pathology-proven HCC at the Liver Cancer Institute of Zhongshan Hospital, Fudan University were tested. They were followed up between October 2004 and November 2010 (72 months). From January 2010 to June 2013, 421 consecutive patients underwent curative resection for HCC by the same surgical team in our department, and 143 patients (cohort 2) were randomly retrieved from a prospectively collected database. Cohort 2 comprised 69 cases who received sorafenib as adjuvant therapy postresection and 74 cases who received only standard-of-care therapy as the control. All the cases were observed until December 2013, with a median observation time of 17.8 months. None received anticancer treatment before surgery or sorafenib administration. The criteria for resectability, specimen collection, and follow-up have been described elsewhere[15, 31-33]. OS and RFS were defined as the interval between the dates of surgery and death, and between the dates of surgery and recurrence, respectively. Treatment modalities after relapse were administered according to uniform guidelines as described previously[31, 34]. If recurrence was not diagnosed, patients were censored on the date of death or the last follow-up.

From June 2011 to June 2013, 114 patients who received sorafenib therapy for core-needle liver biopsy-confirmed advanced HCC in our hospital were retrospectively analyzed. No patients had received previous systemic treatment before sorafenib administration. Among them, the quality of the tumor sample was found to be unreliable in 21 cases, and the tumor response was not evaluable in another 10 cases. Eighty-three cases (cohort 3) with reliable sample quality as well as an evaluable tumor response were examined. Follow-up (every 1.5 months) was completed in December 2013. Patients were required to have at least one untreated target lesion that could be measured in one dimension. Response rate [i.e., complete response (CR), partial response (PR), stable disease (SD), and progressive disease (PD)] was measured according to RECIST (Response Evaluation Criteria in Solid Tumors) guidelines (v1.1)[35] by independent radiologic review. OS and progression-free survival (PFS) were defined as the interval between the dates of administration and death, and between the dates of administration and radiologic progression, respectively. If progression was not verified, patients were censored on the date of death or the last follow-up.

The detailed patient survival is summarized in the Supplemental Materials and Methods. The present study was approved by the appropriate ethics committees, and informed consent was obtained from each patient.

### Tissue microarrays (TMAs):

For cohorts 1 and 2, the postoperative tumor specimens were collected and then constructed into TMAs (Shanghai Biochip Company Ltd, Shanghai, China). TMA construction was performed as described previously[15, 32]. Two 1.0-mm-diameter cores, drilled from each representative formalin-fixed, paraffin-embedded tumor tissue, were sent to make TMA slides. Accordingly, two cylinders from different areas of the tumor samples were obtained, and a total of four TMA chips for cohort 1 and two chips for cohort 2 were prepared.

### Core-needle biopsy:

For cohort 3, the tumor specimens were acquired aseptically through sonographically guided 18-gauge core-needle biopsy of the liver. Next, the samples were collected and made into paraffin sections. All the patients were diagnosed with advanced-stage HCC, as confirmed by independent pathological analysis, before sorafenib administration.

### Immunohistochemistry and evaluation:

Immunohistochemistry in TMAs and other paraffin sections (4-μm thick) was performed by a two-step method as described previously[15, 33]. The primary rabbit monoclonal anti-human HTATIP2 (1:100; Abcam, Cambridge, MA) and mouse monoclonal anti-human CD34 (1:100; Abcam) antibodies were utilized. CD34 was used as a biomarker for vascular endothelial cells, and its immunostaining density was represented by the tumor MVD[15, 16, 36]. The Envision-plus detection system with an anti-rabbit/mouse polymer (Dako, Glostrup, Denmark) was employed. All the sections were stained under the same automation system. Negative controls were treated identically but with omission of the primary antibody.

For all sections, the density of positive staining in whole view was measured using a computerized image system composed of a Leica charge-coupled device camera (DFC500) connected to a Leica DM-IRE2 microscope (Leica, Cambridge, UK)[32]. Briefly, images of five representative fields at ×200 magnification were captured using Leica QWin Plus v3 software, and identical settings were used for each image. For evaluation of HTATIP2 expression and MVD values, the integrated optical density (IOD) and areas of immunostaining in all the images were measured using Image-Pro Plus v6.2 software. A uniform setting for all the sections was applied. Results were quantified as HTATIP2 IOD/total area, and as CD34-positive area/total area.

### Statistical analyses:

Analyses were performed using SPSS 16.0 for Windows. The cutoff point of the HTATIP2 density or MVD for the definition of subgroups (high- versus low-risk groups) was the median value. Pearson's χ^2^ test was used to compare qualitative variables, and quantitative variables were analyzed by *t* or Spearman's test. Data were described by Pearson's correlation coefficient to determine the association between HTATIP2 expression and MVD. Cumulative survival curves were estimated using the Kaplan–Meier method, and differences between the curves were calculated using the log-rank test. Independent prognostic significance of risk factors identified by univariate analysis was computed by the Cox regression model. ROC curve analysis was applied to determine the predictive value among the parameters. Statistical significance was set at a two-sided *P* value<0.05.

## SUPPLEMENTAL MATERIALS AND METHODS


