# Supplementary material for: The combination of HTATIP2 expression and microvessel density predicts converse survival of hepatocellular carcinoma with or without sorafenib
Source: Oncotarget. 2014 May 27;5(11):3895–906. doi: 10.18632/oncotarget.2019 (PMC4116529; doi:10.18632/oncotarget.2019)
Supplement: Supplementary file 1 [file oncotarget-05-3895-s001.pdf]

The combination of HTATIP2 expression and microvessel density predicts converse survival of hepatocellular carcinoma with or without sorafenib

1. SUPPLEMENTAL MATERIALS AND METHODS

1.1. Patient survival:

At the last follow-up, 1, 129 patients had tumor recurrence, and 104 were found to have died. The 1-, 3-, and 5-year overall survival (OS) rates were 87.5%, 66.0%, and 64.9%, respectively; the recurrence rates over those same time intervals were 25.6%, 41.8%, and 43.4%, respectively. At the last follow-up for 69 sorafenib-administered patients in cohort 2, 30 patients had tumor recurrence, and 27 had died. The 1-, 2-, and 4-year OS rates were 91.3%, 76.8%, and 60.9%, respectively; the 1-, 2-, and 4-year recurrence rates were 13.0%, 30.4%, and 43.5%, respectively. For 74 sorafenib non-administration (control) cases, 39 patients had tumor recurrence, and 36 had died. The 1-, 2-, and 4-year OS rates for control patients were 73.9%, 59.4%, and 47.8%, respectively; the 1-, 2-, and 4-year recurrence rates were 31.9%, 49.3%, and 56.5%, respectively.

At the last follow-up for patients in cohort 3, 66 patients had disease progression, and 45 had died. The median OS was 9.2 months (95% confidence interval [CI], 6.8–11.6), the 1-year survival rate was 43%, the median progression-free survival (PFS) was 6.2 months (95% CI, 4.9–7.5), and the estimated rate of PFS at 6 months was 51%. In addition, two patients (2%) had a partial response, 58 (70%) had stable disease, and none achieved a complete response. The disease-control rate (the percentage of patients who had a best-response rating of complete or partial response or stable disease that was maintained for at least 28 days after the first demonstration of that rating on independent radiologic review) was 36.2%.

1.2. Immunohistochemistry:

Immunohistochemical evaluation of proliferating cell nuclear antigen (PCNA) in tissue microarrays (TMAs) of cohort 2 was consistent with the HTATIP2 or CD34 expression and was described in the materials and methods section of the manuscript. The primary rabbit polyclonal anti-human PCNA (1:80; Sigma, St.

Louis, MO) antibody was used. The Image-Pro Plus v6.2 software was adopted to evaluate the positive staining area. The density of PCNA was represented as the index of the integrated optical density (IOD)/total area. The cutoff point of PCNA density for the definition of subgroups was the median value.

1.3. Receiver operating characteristic (ROC) analysis:

In doing the survival analysis, patients were classified into high- and low-risk subgroups according to the HTATIP2 density or microvessel density (MVD) value. When performing ROC analysis, the high- or low-risk group was redefined as the abnormal or normal HTATIP2/MVD group according to their prognosis in survival analysis: the good prognostic indicators were defined as the normal group (high-HTATIP2 expression group or low-MVD group in cohort 1, and low-HTATIP2 group or high-MVD group in sorafenib-administered patients of cohort 2), and the poor prognostic indicators were defined as the abnormal group (low-HTATIP2 group or high-MVD group in cohort 1, and high-HTATIP2 group or low-MVD group in cohort 2).

Table S1: Relationship between the intratumoral HTATIP2/microvessel density and clinicopathological features in cohort 2.

Variables	HTATIP2 density[a]					Microvessel density[a]				
	Low (*n* = 71)		High (*n* = 72)			Low (*n* = 71)		High (*n* = 72)		
	No. of patients	%	No. of patients	%	*P*	No. of patients	%	No. of patients	%	*P*
Age, years					.669					.562
≤ 50	31	44	34	47		34	48	31	43	
> 50	40	56	38	53		37	52	41	57	
Gender					.973					.139
Male	61	86	62	86		58	82	65	90	
Female	10	14	10	14		13	18	7	10	
Hepatitis B history					.802					.505
Yes	56	79	58	81		55	78	59	82	
No	15	21	14	19		16	22	13	18	
HBeAg					.470					.112
Positive	16	22	20	28		22	31	14	19	
Negative	55	78	52	72		49	69	58	81	
ALT, U/L					.530					.980
≤ 75	64	90	67	93		65	92	66	92	
> 75	7	10	5	7		6	8	6	8	
AFP, ng/dl					.015					.054
≤ 300	25	35	40	56		38	54	27	37	
> 300	46	65	32	44		33	46	45	63	
Liver cirrhosis					.618					.972

Yes	59	83	62	86		60	85	61	85	
No	12	17	10	14		11	15	11	15	
Tumor size, cm					.002					.008
≤ 5	32	45	51	71		49	69	34	47	
> 5	39	55	21	29		22	31	38	53	
Tumor differentiation					.078					.055
High (Stage I–II)	43	61	33	46		32	45	44	61	
Low (Stage III–IV)	28	39	39	54		39	55	28	39	
Intrahepatic metastasis					.006					.009
Yes	41	58	25	35		25	35	41	57	
No	30	42	47	65		46	65	31	43	
Tumor encapsulation					.091					.322
Complete	20	28	30	42		22	31	28	39	
No	51	72	42	58		49	69	44	61	
Microvascular invasion					.000					.001
Yes	37	52	17	24		17	24	37	51	
No	34	48	55	76		54	76	35	49	
TNM stage					.027					.020
I	6	8	12	17		11	16	7	10	
II	27	38	37	51		38	53	26	36	
IIIA	38	54	23	32		22	31	39	54	

[a]The HTATIP2 density (mean ± standard deviation) was 0.0696 ± 0.0519 (median, 0.0594; range, 0.000223–0.495), and the microvessel density was 0.0905 ± 0.0962 (median, 0.0736; range, 0.00234–0.505).

$P < 0.05$ was considered statistically significant.

Abbreviations: HTATIP2, HIV-1 Tat interactive protein 2; HBeAg, hepatitis B e antigen; ALT, alanine aminotransferase; AFP, α-fetoprotein; TNM, tumor-node-metastasis.

Table S2: Univariate and multivariate analyses for the survival and recurrence of 69 sorafenib-administered patients in cohort 2.

	Overall survival				Recurrence-free surviva		
	Univariate *P*	Multivariate			Univariate *P*	Multivaria	
		HR	95% CI	*P*		HR	95% CI
> 50 years	.176			NA	.330		
le *vs* male	.137			NA	.666		
story: no *vs* yes	.661			NA	.616		
tive *vs* positive	.125			NA	.425		
s: no *vs* yes	.988			NA	.786		
> 75 U/L	.614			NA	.850		
s > 300 ng/dl	.031			NS	.848		
5 *vs* > 5 cm	.212			NA	.248		
entiation: low *vs* high	.120			NA	.260		
er: single *vs* multiple	.870			NA	.224		
netastasis: no *vs* yes	.568			NA	.120		
sulation: no *vs* complete	.443			NA	.866		
r invasion: no *vs* yes	.093			NA	.189		
vs II *vs* IIIA	.877			NA	.467		
y: low *vs* high	.013			NS	.028		
nsity: low *vs* high	< .001	4.567	1.867–11.172	.001	.001	4.165	1.896–9.150
ensity: low *vs* high	.001	0.254	0.104–0.621	.003	.002	0.444	0.209–0.942
ATIP2 and microvessel density	< .001			NA	< .001		

Abbreviations: HR, hazard ratio; CI, confidence interval; NA, not adopted; HBeAg, hepatitis B e antigen; ALT, alanine aminotransferase; AFP, α-fetoprotein; NS, not significant; TNM, tumor-node-metastasis; PCNA, proliferating cell nuclear antigen; HTATIP2, HIV-1 Tat interactive protein 2.

Table S3: Prognostic values of variables for death and 1-year tumor recurrence by ROC analysis in cohort 1.

Variables	Area under curve	95% CI	*P*
Death			
Tumor size	0.605	0.538–0.672	.003
Tumor differentiation	0.575	0.506–0.645	.032
Intrahepatic metastasis	0.596	0.526–0.666	.006
TNM stage	0.694	0.632–0.756	< .001
HTATIP2 density	0.690	0.625–0.754	.001
Microvessel density	0.706	0.644–0.767	< .001
Combined HTATIP2 and microvessel density	0.730	0.669–0.790	< .001
1-year recurrence			
HBeAg	0.560	0.494–0.626	.078
Tumor size	0.585	0.520–0.651	.012
Intrahepatic metastasis	0.571	0.504–0.637	.037
TNM stage	0.603	0.538–0.668	.002
HTATIP2 density	0.582	0.516–0.647	.016
Microvessel density	0.591	0.526–0.656	.007
Combined HTATIP2 and microvessel density	0.690	0.619–0.759	< .001

Abbreviations: ROC, receiver operating characteristic; CI, confidence interval; TNM, tumor-node-metastasis; HTATIP2, HIV-1 Tat interactive protein 2; HBeAg, hepatitis B e antigen.

Table S4: Prognostic values of variables for death and tumor recurrence by ROC analysis of 69 sorafenib-administered patients in cohort 2.

Variables	Area under curve	95% CI	*P*
Death			
PCNA density	0.624	0.488–0.760	.083
HTATIP2 density	0.751	0.629–0.873	< .001
Microvessel density	0.739	0.616–0.863	.001
Combined HTATIP2 and microvessel density	0.848	0.752–0.945	< .001
Recurrence			
PCNA density	0.608	0.472–0.743	.127
HTATIP2 density	0.705	0.578–0.832	.004
Microvessel density	0.633	0.500–0.767	.049
Combined HTATIP2 and microvessel density	0.754	0.636–0.872	< .001

Abbreviations: ROC, receiver operating characteristic; CI, confidence interval; PCNA, proliferating cell nuclear antigen; HTATIP2, HIV-1 Tat interactive protein 2.

Table S5: Summary of efficacy measures based on the HTATIP2/microvessel density in cohort 3.

Overall response[b]	HTATIP2 density[a]					Microvessel density[a]				
	Low (*n* = 41)		High (*n* = 42)		*P*	Low (*n* = 41)		High (*n* = 42)		*P*
	No. of patients	%	No. of patients	%		No. of patients	%	No. of patients	%	
CR	0	0	0	0	.004	0	0	0	0	.037
PR	2	5	0	0		0	0	2	5	
SD	34	83	24	57		25	61	33	78	
PD	5	12	18	43		16	39	7	17	
NE[c]	7	–	3	–	–	4	–	6	–	–

[a]The HTATIP2 density (mean ± standard deviation) was 0.0721 ± 0.0854 (median, 0.0626; range, 0.000187–0.494, and the microvessel density was 0.0950 ± 0.0868 (median, 0.0756; range, 0.00146–0.485).

[b]Measured according to RECIST (Response Evaluation Criteria in Solid Tumors) guideline (v1.1, reference 34) by independent radiologic review. The data presented were recorded based on the first evaluation of tumor response.

[c]Not including cohort 3 (83 cases). The quality of the tumor sample is reliable, whereas the level of response is nonevaluable.

$P < 0.05$ was considered statistically significant.

Abbreviations: HTATIP2, HIV-1 Tat interactive protein 2; CR, complete response; PR, partial response; SD, stable disease; PD, progressive disease; NE, nonevaluable.

Supplemental figure legends:

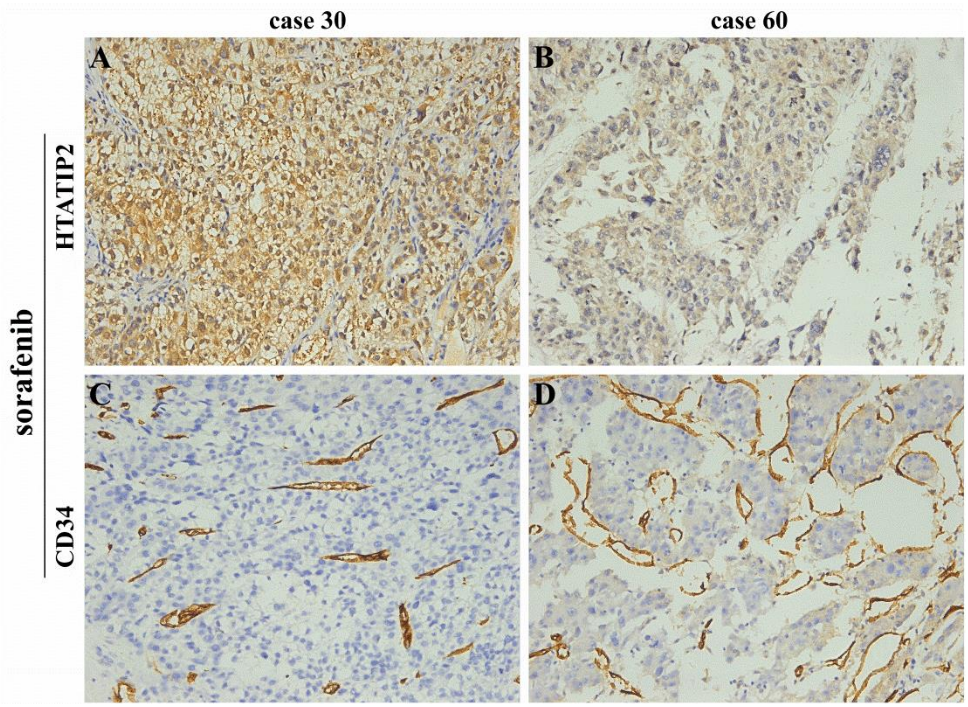

Figure S1: Images of representative high or low HTATIP2 expression and microvessel density (MVD, indicated by CD34) determined by immunostaining in paraffin sections from cohort 3. (A and C) Case 30 showed high HTATIP2 expression but low MVD, whereas, (B and D) case 60 showed low HTATIP2 expression but high MVD (×200).

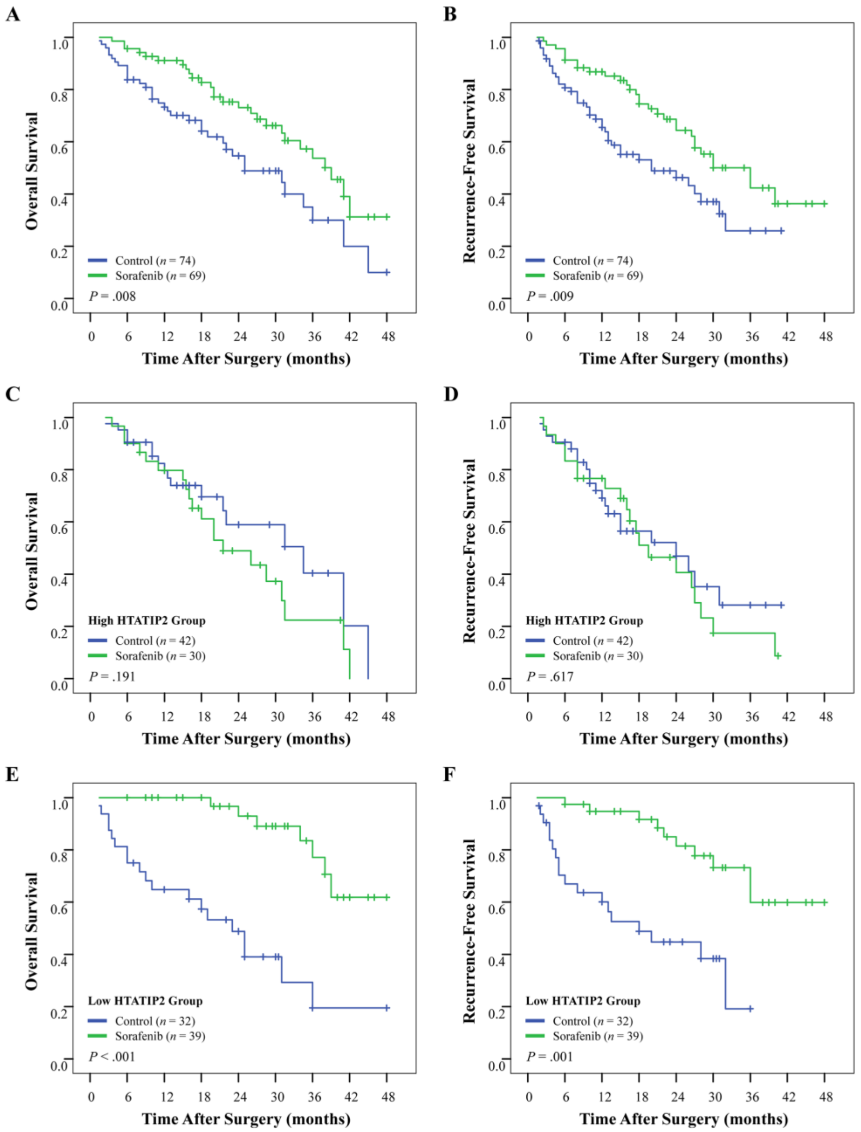

Figure S2: Cumulative overall survival (OS) and recurrence-free survival (RFS) curves of 143 patients with high or low expression of HTATIP2 in cohort 2. (A and B) Sorafenib prolonged postoperative OS and RFS compared with the control. (C and D) Sorafenib did not impact OS and RFS in the HTATIP2 high-expression group. (E and F) Sorafenib significantly prolonged OS and RFS in HTATIP2 low-expression group.

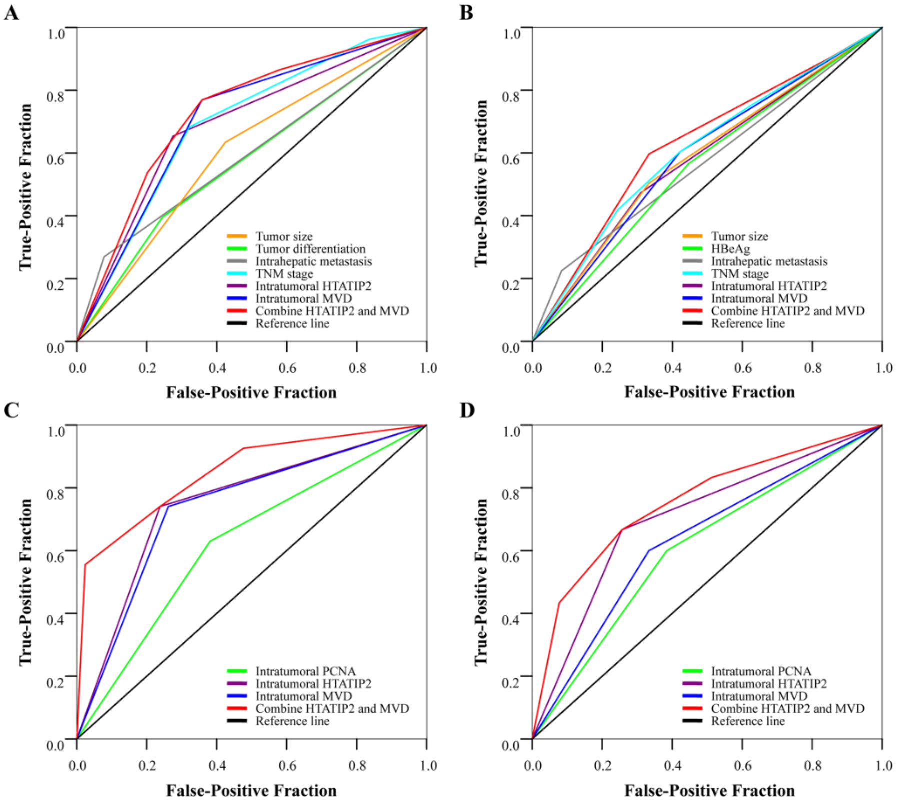

Figure S3: Receiver operating characteristic (ROC) analysis curve. Clinicopathological factors identified by multivariate analysis and the combination of HTATIP2 expression and microvessel density (MVD) were adopted (see the detailed definition of the classification of factors in the supplemental materials and methods section). The combination of HTATIP2 expression and MVD precisely predicted death and recurrence for both (A and B) cohort 1 and (C and D) sorafenib-administered patients in cohort 2 ($P < 0.001$ for all), and its predictive value was the best among all of the adopted factors.
